# Anent Medical Journals

**Published:** 1879-02

**Authors:** 


### Editorial.

### ARTICLE XV.

### ANENT MEDICAL JOURNALS.

The second semi-centennial of the existence of the *Boston Medical and Surgical Journal* is fitly begun by the publication of an interesting and valuable communication by Dr. J. S. Billings, of the U. S. Army, on the subject of the medical journals of the United States. The article fills nearly fourteen pages of type, and some idea of its character may be gained from the fact that ten of these pages, in small type, are fully occupied by a complete list of all the medical journals that have ever been published in the country, arranged according to States. The history of medical journalism in Illinois is almost the history of the varying fortunes of the CHICAGO MEDICAL JOURNAL AND EXAMINER, as may be seen from the following extract:

"ILLINOIS.—The *Illinois Medical and Surgical Journal*. Monthly. Edited by J. V. Z. Blaney. Chicago. Vols. I.–II.; 1844–46. 8vo. Continued as the following: The *Illinois and Indiana Medical and Surgical Journal*. Bi-monthly. Edited by J. V. Z. Blaney, D. Brainard and others. Chicago. Vols. I.–II. 1846–1848. 8vo. Continuation of the preceding and continued as the following: The *Northwestern Medical and Surgical Journal*. Bi-monthly. Edited by W. B. Herrick and J. Evans. Chicago. Vols. V.–XIV. 1848–57. 8 vo. Continuation of the preceding and continued as the following: The *Chicago Medical Journal*. Monthly. Edited by N. S. Davis and W. H. Byford. Chicago. Vols. XV.–XXXI, 1858–1875. 8vo. (*) Continuation of the preceding and consolidated with the *Medical Examiner*, Chicago, forming the following: Vols. XXV. and XXVI. Semi-monthly. THE CHICAGO MEDICAL JOURNAL AND EXAMINER. Edited by W. H. Byford and others. Chicago. Vols. XXXII–XXXVII. 1875–1878. 8vo. Current. Formed by the consolidation of the preced-

* Between the years 1858 and 1875 many changes occurred in the editorial staff. At the date of the consolidation in 1875, the *Chicago Medical Journal* was edited by Prof. J. Adams Allen, assisted by Dr. Walter Hay, late of this city.—[ED.]

ing with the *Chicago Medical Examiner*. The *Northwestern Medical Intelligencer*. Bi-monthly. Chicago. 1851. 8 vo. This alternated with the *Northwestern Medical and Surgical Journal*, of which it formed a part. The *Chicago Medical Examiner*. Monthly. Edited by N. S. Davis and E. A. Steele. Chicago. Vols. I.–XII. 1860–1871. 8 vo. Continued as the following: The *Medical Examiner*. Chicago. Semi-monthly. Edited by N. S. Davis and F. H. Davis. Vols. XIII.–XVI. 1872–1875. 4to. In September, 1875, united with the *Chicago Medical Journal*, forming THE CHICAGO MEDICAL JOURNAL AND EXAMINER."

The other journals of the State are registered as follows:

"The *Military Tract Medical Reporter*, L. S. & C. A. Lambert. Galesburg, Ill. Prospectus issued in August, 1871, but the journal never appeared. The *Chicago Journal of Nervous and Mental Disease*. Edited by J. S. Jewell and others. Chicago. Vols. I.–II. 1874–1875. 8vo. Continued as the following: The *Journal of Nervous and Mental Disease*. Edited by J. S. Jewell and others. Chicago. Vols. I–III. New series 1876–1878. 8vo. Continuation of the preceding. *The Medical Register and Advertiser*. Quarterly. Edited by J. I. Hale. Anna, Ill. Nos. 1–2. Vol. 1. 1875. 8vo. The *Monthly Journal of the Southern Illinois Medical Association*. Edited by C. W. Dunning and H. Wardner. Cairo. Vols. I–II. 1877–'78. Vol. I. in six numbers. Current. The *Illinois Medical Recorder*. Monthly. Edited by R. E. Beech. Published under the auspices of the District Medical Society of Central Illinois. Nos. 1–6. June to November. 1878. 8vo. Current. The *American Medical Review and Index*. Monthly. James I. Hale, editor. Anna, Ill. Nos. 1–3. July to September. 1878. 8vo. Current."

Looking at the table of mortality statistics of medical journals throughout the country, we find that of 247 titles commenced (in 1,630 volumes), in ten cases only one number was issued; in twenty-one cases the first volume was never completed; in sixty-one cases there was no survival beyond the first volume; in thirty-nine there was no survival beyond the second volume; and but fifty-three journals of the entire number are now current. These facts furnish in themselves a fertile commentary upon the reasons for which many of the smaller journals exist—journals which have been published,—"not for direct profit, but as an indirect advertisement, or—and this is more common—the desire to have a place in which the editor can speak his mind and attack his enemies without restraint."

We could wish that Dr. Billings, or some other writer of equal ability, might have the courage to further divide the fifty-three survivors into two classes—the one to represent the journals which are conducted with a view solely to the advancement of science, the others to include those which are the pure and simple advertisements of individuals, medical schools, or dealers in drugs. Though we have often referred to the subject before in these pages, we venture to repeat here that the real prosperity of THE CHICAGO MEDICAL JOURNAL AND EXAMINER dates from the hour when its management passed into the hands of the body of the profession in this city, and its two predecessors ceased to be the organs of the two leading medical schools of Chicago. It is a curious commentary on this divorce, that the medical colleges referred to have actually benefited by the change. The stock of the Chicago Medical Press Association is held by eighty-three prominent professional gentlemen of this city, who represent every institution and phase of medical interest.

We have carefully perused the list of the fifty-three surviving journals and have deliberately concluded that but from fourteen to twenty are of any permanent value to the profession, and worthy of general circulation or to be sent abroad and handed down to posterity as fit exponents of American medical periodical literature. Some of the others possess no value whatever apart from the gleanings which they regularly make from the crops carefully harvested by their more thrifty neighbors. Some are absolutely worthless, judged from any stand-point, and a few are undeniably worse than worthless—they are dangerous and disgusting parasites upon the body medical.

Of this latter class are the cheap publications which aim to be classed as medical journals, but which are really the clumsily disguised advertising sheets of certain pretentious drug-houses of doubtful reputation. Some of these have lately embarked in the business of attempting to add several doubtful "novelties" to the *ruda indigestaque moles* of the drug lists. Though exposed in one instance, an exposure which it is to be hoped will not be without effect upon the others, one can still see in their pages brief certificates from "physicians" in various parts of the country as to the singular value of this or that "remedy." The names of these endorsers are usually unknown in any other connection, and will be vainly sought among the list of contributors to really scientific periodicals. Some of them, we blush to say it, have pushed their trashy reports into the proceedings of

State Societies. Others figure as authors in the absurdly incongruous Materia Medica lists of the homœopathic journals.

The sterling common sense of the mass of the physicians throughout the country must supply the remedy for this evil. We call upon every good man, who values his own reputation, and who cares for the elevation of the standards of the profession he has chosen, to contribute money for the support of the best medical journals, and of these only. In this connection we do not ask such to subscribe for this particular journal. The subject is one of such importance that if the desired result could be completely attained by the suppression of this periodical, we would gladly make the sacrifice in the interest of the profession at large. But the MEDICAL JOURNAL AND EXAMINER happens to be one of the very few publications of the country that is neither a piece of private property nor an organ. It has advanced to the first rank of American journals, by the observation of the very principles which are now emphasized. Content with its own position and circulation, it urges the medical men of the United States to cease contributing to the support of the weakly publications that eke out a miserable existence on the pabulum of the advertisement.

It is the duty and interest of every physician in the land to support heartily the best of our journals, and none other. Let it be understood that it is a stigma upon one's reputation, that his name has appeared in an obscure advertising sheet in connection with the endorsement of a questionable "novelty" in drugs, or even as an author. And we call upon the editors of the respectable medical journals of America to refuse an exchange with every periodical of doubtful character and provincial position. That will be a bright day for medical journalism in the United States, which sees the organization of an Associated Medical Press, membership to which will be restricted to the managing editors of really good journals in existence, the privilege of membership and exchange to be granted only by unanimous consent of those already in enjoyment of these privileges. Under such an arrangement, advertising firms and book publishers might cease to offer a premium for mediocrity and worthlessness in the periodical medical literature of our land.

